# Agronomical and analytical trait data assessed in a set of quinoa genotypes growing in the UAE under different irrigation salinity conditions

**DOI:** 10.1016/j.dib.2020.105758

**Published:** 2020-05-30

**Authors:** Fatima Zahra Rezzouk, Mohammad Ahmed Shahid, Ismahane A. Elouafi, Bangwei Zhou, José L. Araus, Maria D. Serret

**Affiliations:** a Section of Plant Physiology, University of Barcelona, 08028 Barcelona, and AGROTECNIO (Center of Research in Agrotechnology), 25198 Lleida, Spain.; b International Center for Biosaline Agriculture (ICBA), P.O. Box 14660, Dubai, U.A.E.; c Key Laboratory of Vegetation Ecology, Ministry of Education, Institute of Grassland Science, Northeast Normal University, Changchun, China.

**Keywords:** Irrigation, Isotopic composition, Leaf pigments, Mineral content, Manuring, Quinoa, Seed yield

## Abstract

The importance of quinoa has been emphasized considerably in the recent decades, as a highly nutritional crop seed that is tolerant to salinity and amenable to arid agronomical conditions. The focus of this paper is to provide raw and a supplemental data of the research article entitled “Agronomic performance of irrigated quinoa in desert areas: comparing different approaches for early assessment of salinity stress” [1], aiming to compare different approaches for early detection, at the genotypic and crop levels, of the effect of salinity caused by irrigation on the agronomic performance of this crop. A set of 20 genotypes was grown under drip irrigation in sandy soil, amended with manure, at the International Center for Biosaline Agriculture (UAE) for two weeks, after which half of the trial was submitted to irrigation with saline water and this was continued until crop maturity. After eight weeks of applying the two irrigation regimes, pigment contents were evaluated in fully expanded leaves. The same leaves were then harvested, dried and the stable carbon and nitrogen isotope compositions (δ^13^C and δ^15^N) and the total nitrogen and carbon contents of the dry matter analyzed, together with ion concentrations. At maturity yield components were assessed and yield harvested. Data analysis demonstrated significant differences in genotypes response under each treatment, within all assessed parameters. The significant level was provided using the Tukey-b test on independent samples. The present dataset highlights the potential use of different approaches to crop phenotyping and monitoring decision making.

## Value of the Data

• The data presented here provides a reference to the physiological and agronomical performance of a wide pool of quinoa accessions grown under different salinity conditions in desert areas.
• This dataset is useful for researchers involved in agronomy and breeding for saline agriculture in arid regions, with particular focus on quinoa and manuring.
• This dataset includes a wide range of phenotypical and agronomical traits that may be used to develop more efficient experimental set ups and refine simulation models, including crop management and phenotyping protocols.
• Quinoa is a very promising crop that is amenable to arid zones affected by salinity, which makes it a real alternative for current application in agriculture and it has great potential as a crop in the context of climate change. It may provide a source of income to farmers in developing countries.

Specifications Table

**Table:** 

Subject	Agronomy and Crop Science
Specific subject area	This dataset provides information comparing a wide range of approaches for early assessment of salinity stress in quinoa under irrigation and the negative effect of excessive manuring.
Type of data	TablesFigure
How data were acquired	Leaf pigments were assessed using a portable leaf-clip sensor (Dualex, Dualex Force-A, Orsay, France). The Dualex sensor operates with a UV excitation beam at 357 nm, which corresponds to the maximum absorption for flavonoids, and a red reference beam at 650 nm, which corresponds to the maximum absorption for chlorophyll [2]. Stable isotopic composition of leaf dry matter were acquired by pulverizing dried leaf samples using a Mixer Mill (MM400, RETSCH GmbH, Germany) and subsampling approximately 1 mg of the pulverized material into tin capsules for further analysis using an elemental analyzer (Flash 1112 EA; ThermoFinnigan, Schwerte, Germany) coupled with an isotope ratio mass spectrometer (Delta C IRMS, ThermoFinnigan), operating in continuous flow mode. Soluble fraction was determined by subsampling 50 mg of the pulverized leaf material and suspending each sample with 1 mL of Milli-Q water in an Eppendorf tube (Eppendorf Scientific, Hamburg, Germany) for 20 min at about 5°C. The sample was then centrifuged at 12000 g for 5 min and at 5°C. Afterwards, the supernatant containing the water-soluble fraction was pipetted into a new Eppendorf and heated at 100°C for 3 min to denature the proteins. Samples were centrifuged again (12000 g for 5 min at 5°C), and 100 µl of the resulting aliquot was placed in tin capsules and dried at 70°C for 2 hours. The soluble fraction of carbon and nitrogen isotope compositions was then determined in the same manner as the stable isotopic composition of the leaf dry matter. Ion concentrations in leaves were obtained by acid-digesting and diluting 100 mg of each sample; then the solution was analyzed using an Inductively Coupled Plasma Emission Spectrometer (L3200RL, Perkin Elmer, Uberlingen, Germany).
Data format	Raw Analyzed
Parameters for data collection	Leaf pigment contents were determined around 8 weeks after the two irrigation treatments were imposed. Afterwards, the same leaves were washed with tap and distilled water, dried in an oven at 60°C for 48h, and ground to a fine powder for further ion and stable isotopic composition and total N and C analyses.
Description of data collection	Pigments were measured in 10 fully expanded leaves, selected from the central rows. At physiological maturity, 5 plants were selected from the central rows. Height was measured from the ground to the top of the inflorescence, and number of branches was recorded at different node positions. Number of inflorescences per plant was counted, and the length of 3 random inflorescences was averaged. Biomass and seed yield were assessed by manually harvesting the 5 plants from the middle row of each plot. Ion and stable isotopic composition were analyzed at the Scientific Facilities of the University of Barcelona Max, min and average temperature, and precipitation data were acquired from the meteorological station at ICBA.
Data source location	Institution: International Center for Biosaline Agriculture (ICBA) City: Dubai Country: The United Arab Emirates Latitude and longitude (and GPS coordinates) for collected samples/data: 25°05′49′′ N and 55°23′25′′E
Data accessibility	Repository name: Mendeley Data DOI: 10.17632/r5ywtt8w39.1 (reserved but not active until publication) Direct URL to data: https://data.mendeley.com/datasets/r5ywtt8w39/draft?a=fb0d4661-eaf5-4781-80a5-0913bba85cb5
Related research article	Fatima Zahra Rezzouk, Mohammad Ahmed Shahid, Ismahane A. Elouafi, Bangwei Zhou, José L. Araus, Maria D. Serret, Agronomic performance of irrigated quinoa in desert areas: comparing different approaches for early assessment of salinity stress Agricultural Water Management

## Data description

Supplemental tables displaying averaged values of yield components (supplemental table 1), ion concentrations (supplemental table 2), pigments (supplemental table 3), stables isotopes and their elemental analysis (supplemental table 4), of quinoa accessions grown under different irrigation treatments (fresh water and saline water), and genotypes (20 lines), exhibiting significant differences between treatments and among genotypes. Thus, means exhibiting different letters are significantly different (P < 0.05) by the post-hoc Tukey-b test on independent samples within each treatment (Fresh water and saline water). Values for accessions 10 and 18 under saline irrigation conditions are not included in the median separation because of the lack of replications. The distribution of climate parameters (maximum, minimum and average temperatures, and precipitation) during the quinoa growing period is displayed in supplemental Fig. 1.

**Fig. 1:**
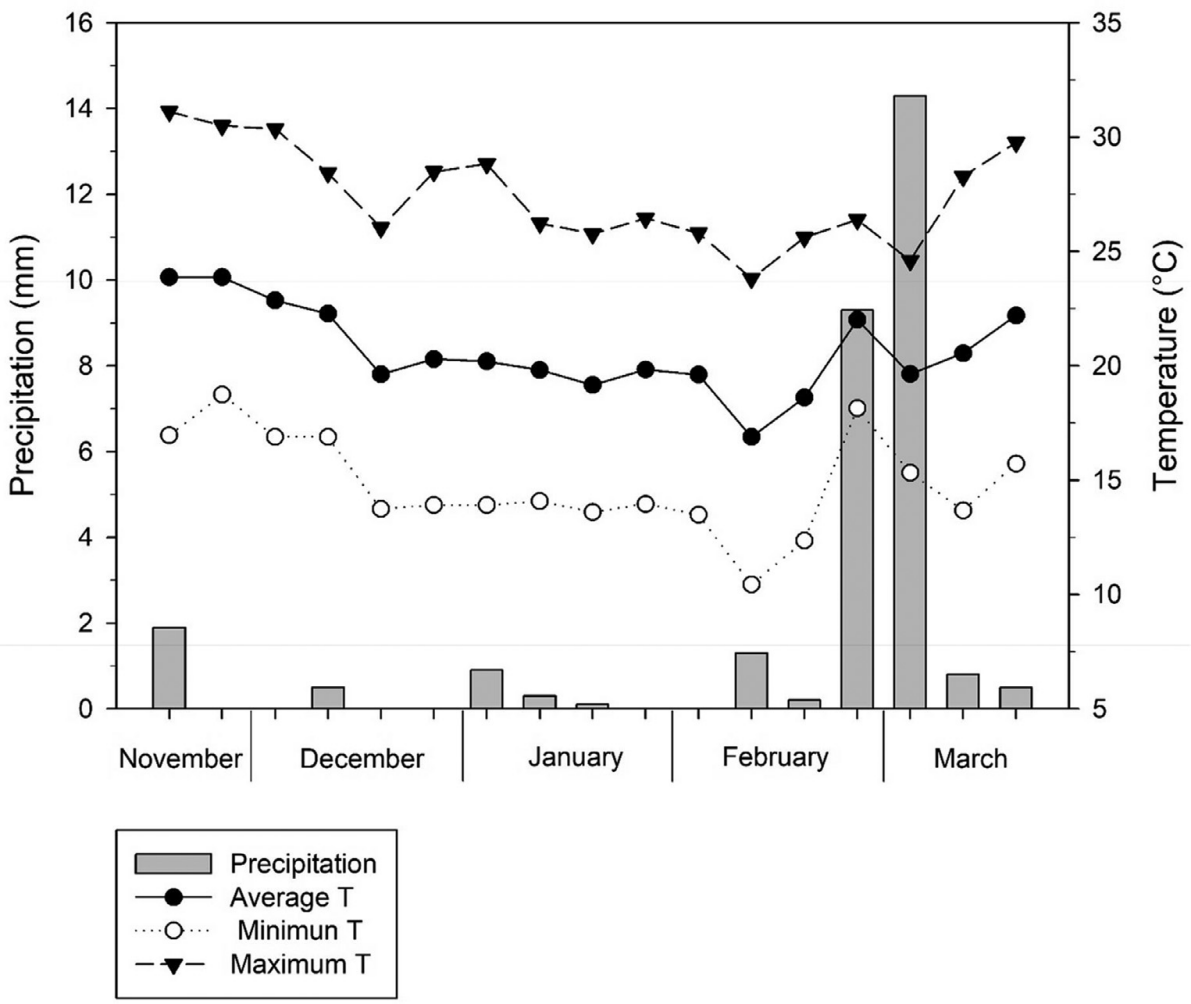
Maximum, minimum and average temperature and precipitation during the quinoa growing period.

For each trait, the values provided correspond to the three replicates per genotype and the two irrigation (fresh water and saline water) treatments. Assessed traits were: yield components (seed yield, biomass, plant height, branches, inflorescences, inflorescence length) at maturity, together with ion concentrations (sodium, phosphorus, potassium, calcium, magnesium concentrations and the K^+^/Na^+^, Ca^2+^/Na^+^ and Mg^2+^/Na^+^ ratios), leaf pigments (chlorophylls, flavonoids, anthocyanins and nitrogen balance index (NBI)), carbon and nitrogen concentrations on a dry matter basis, and carbon (δ^13^C) and nitrogen (δ^15^N) isotope composition in the dry matter and soluble fraction measured in fully expanded leaves 8 weeks after irrigation treatments were imposed are presented in the Raw data Tables 1, 2, 3 and 4.

**Table 1:** Average plant height, branches per plant, inflorescences per plant, inflorescence length, biomass and seed yield in the set of quinoa accessions grown under fresh water and saline irrigation treatments. Means exhibiting different letters are significantly different (P < 0.05) by the post-hoc Tukey-b test on independent samples within each treatment (fresh and saline water). Values for accessions 10 and 18 are presented but not included in the separation of means because of their poor agronomical performance, particularly under saline irrigation. Genotype numbers as detailed in Table 1.

Treatment	Genotype	Yield components					
		Plant height (cm)	Branches plant^−1^	Inflorescences plant^−1^	Inflorescence length (cm)	Biomass (g m^−2^)	Seed yield (g m^−2^)

*Fresh water*	1	102.6**^bc^**	8.27**^ab^**	7.60**^ab^**	26.90**^cde^**	2225**^ab^**	504.8**^a^**
	2	123.8**^ab^**	7.67**^ab^**	6.87**^ab^**	39.90**^b^**	1960**^ab^**	459.3**^a^**
	3	144.8**^a^**	4.60**^b^**	4.00**^b^**	50.83**^a^**	2680**^ab^**	392.2**^ab^**
	4	122.1**^b^**	6.93**^ab^**	5.93**^ab^**	38.48**^bc^**	2060**^ab^**	400.7**^ab^**
	5	88.2**^cd^**	6.00**^b^**	5.53**^ab^**	27.90**^bcde^**	1427**^b^**	297.0**^ab^**
	6	88.9**^cd^**	9.13**^ab^**	7.87**^ab^**	33.07**^bcde^**	2460**^ab^**	428.4**^ab^**
	7	100.6**^bcd^**	7.27**^ab^**	6.53**^ab^**	35.03**^bcd^**	2000**^ab^**	510.0**^a^**
	8	120.3**^b^**	7.67**^ab^**	5.20**^ab^**	40.13**^b^**	1940**^ab^**	544.3**^a^**
	9	114.9**^b^**	7.47**^ab^**	4.80**^b^**	33.77**^bcde^**	1400 **^b^**	401.3**^ab^**
	10	46.89^−^	7.13^−^	6.87^−^	17.99^−^	690^−^	42.15^−^
	11	111.7**^bc^**	8.07**^ab^**	6.33**^ab^**	34.77**^bcd^**	2920**^ab^**	402.4**^ab^**
	12	117.9**^b^**	11.07**^a^**	9.07**^a^**	39.07**^bc^**	3080**^ab^**	440.3**^a^**
	13	107.8**^bc^**	8.93**^ab^**	8.13**^ab^**	35.77**^bcd^**	3440**^a^**	543.6**^a^**
	14	110.1**^bc^**	8.27**^ab^**	7.00**^ab^**	33.37**^bcde^**	2080**^ab^**	363.3**^ab^**
	15	61.8**^ef^**	7.80**^ab^**	6.73**^ab^**	23.97**^de^**	2180**^ab^**	323.4**^ab^**
	16	53.8**^f^**	6.33**^ab^**	5.87**^ab^**	22.20**^e^**	1483**^b^**	84.7**^b^**
	17	111.3**^bc^**	8.40**^ab^**	7.33**^ab^**	33.93**^bcde^**	2685**^ab^**	632.4**^a^**
	18	25.37^−^	5.73^−^	5.47^−^	10.53^−^	464^−^	50.8^−^
	19	104.5**^bc^**	7.60**^ab^**	7.13**^ab^**	32.97**^bcde^**	2120**^ab^**	503.0**^a^**
	20	77.2**^de^**	8.73**^ab^**	7.87**^ab^**	29.37**^bcde^**	1395**^b^**	354.4**^ab^**
*Saline water*	1	84.6**^bc^**	6.73**^b^**	6.07**^b^**	25.53**^cdefg^**	1487**^bc^**	386.2**^abc^**
	2	85.7**^bc^**	6.73**^b^**	5.73**^b^**	28.00**^bcdef^**	1140**^bc^**	187.3**^cd^**
	3	115.1**^a^**	6.07**^b^**	5.33**^b^**	39.53**^a^**	1940**^abc^**	221.5**^bcd^**
	4	106.5**^ab^**	10.3**^a^**	8.80**^a^**	35.67**^ab^**	1700**^bc^**	249.0**^abcd^**
	5	78.5**^c^**	6.67**^b^**	5.80**^b^**	30.60**^bcde^**	1410**^bc^**	216.8**^bcd^**
	6	80.9**^bc^**	5.40**^b^**	5.40**^b^**	28.88**^bcde^**	1610**^bc^**	442.4**^a^**
	7	65.7**^cd^**	4.80**^b^**	4.60**^b^**	27.37**^bcdefg^**	1300**^bc^**	286.9**^abcd^**
	8	88.9**^bc^**	6.33**^b^**	5.80**^b^**	30.37**^bcde^**	1620**^bc^**	379.5**^abc^**
	9	85.0**^bc^**	6.07**^b^**	5.60**^b^**	31.40**^bcd^**	1380**^bc^**	289.8**^abcd^**
	10	31.58^−^	2.90^−^	2.80^−^	14.5^−^	-	-
	11	91.4**^bc^**	7.53**^b^**	7.53**^ab^**	33.80**^bcd^**	3480**^a^**	416.1**^ab^**
	12	47.9**^d^**	6.47**^b^**	6.13**^b^**	24.57**^defg^**	1707**^bc^**	107.4**^d^**
	13	83.9**^bc^**	6.13**^b^**	6.13**^b^**	32.50**^bcd^**	2660**^abc^**	281.8**^abcd^**
	14	80.9**^bc^**	6.80**^b^**	6.53**^ab^**	34.37**^abc^**	2200**^abc^**	380.1**^abc^**
	15	52.7**^d^**	5.27**^b^**	4.67**^b^**	21.80**^efg^**	1242**^bc^**	198.3**^cd^**
	16	45.0**^d^**	6.80**^b^**	5.53**^b^**	18.90**^g^**	2967**^ab^**	226.8**^bcd^**
	17	82.6**^bc^**	6.40**^b^**	5.67**^b^**	27.87**^bcdef^**	1715**^bc^**	387.5**^abc^**
	18	21.17^−^	2.80^−^	2.67^−^	9.55^−^	1350^−^	51.8^−^
	19	66.2**^cd^**	6.07**^b^**	5.80**^b^**	26.30**^cdefg^**	1060**^c^**	280.1**^abcd^**
	20	44.1**^d^**	4.93**^b^**	4.60**^b^**	19.28**^fg^**	1040**^c^**	188.6**^bcd^**

**Table 2:** Average sodium, phosphorus, potassium, calcium and magnesium concentrations and the K^+^/Na^+^, Ca^2+^/Na^+^ and Mg^2+^/Na^+^ ratios in fully expanded leaves of quinoa accessions grown for eight weeks under different (fresh water and saline) irrigation treatments. Means exhibiting different letters are significantly different (P < 0.05) by the post-hoc Tukey-b test on independent samples within each treatment (fresh and saline water). Values for accession 18 under saline irrigation conditions are not included in the median separation because of the lack of replications. Genotype numbers as detailed in Table 1.

Treatment	Genotype	Ion concentrations			Ratios				
		Na^+^(mmol.g^−1^)	P (mmol.g^−1^)	K^+^(mmol.g^−1^)	Ca^2+^(mmol.g^−1^)	Mg^2+^ (mmol.g^−1^)	K^+^/Na^+^	Ca^2+^/Na^+^	Mg^2+^/Na^+^

*Fresh water*	1	0.05**^a^**	0.16**^bc^**	1.61**^a^**	0.48**^ef^**	0.32**^f^**	30.20**^ab^**	8.99**^a^**	5.95**^a^**
	2	0.03**^a^**	0.15**^bc^**	1.65**^a^**	0.49**^ef^**	0.36**^ef^**	64.06**^ab^**	19.21**^a^**	13.92**^a^**
	3	0.04**^a^**	0.18**^ab^**	1.53**^a^**	0.49**^ef^**	0.43**^bcdef^**	45.69**^ab^**	14.37**^a^**	13.12**^a^**
	4	0.06**^a^**	0.19**^ab^**	1.57**^a^**	0.59**^bcdef^**	0.42**^cdef^**	44.17**^ab^**	14.77**^a^**	10.10**^a^**
	5	0.07**^a^**	0.09**^bc^**	1.50**^a^**	0.84**^ab^**	0.62**^bc^**	23.41**^ab^**	13.03**^a^**	9.57**^a^**
	6	0.14**^a^**	0.05**^c^**	2.03**^a^**	0.78**^abcd^**	0.42**^cdef^**	16.72**^ab^**	6.19**^a^**	3.31**^a^**
	7	0.08**^a^**	0.11**^bc^**	1.98**^a^**	0.67**^bcdef^**	0.61**^bc^**	25.90**^ab^**	8.49**^a^**	7.78**^a^**
	8	0.05**^a^**	0.18**^ab^**	1.44**^a^**	0.53**^def^**	0.36**^ef^**	29.51**^ab^**	11.02**^a^**	7.45**^a^**
	9	0.05**^a^**	0.17**^abc^**	1.45**^a^**	0.81**^abc^**	0.52**^bcdef^**	34.66**^ab^**	17.92**^a^**	12.12**^a^**
	10	0.08**^a^**	0.09**^bc^**	1.95**^a^**	0.70**^bcdef^**	0.68**^ab^**	25.78**^ab^**	9.16**^a^**	8.97**^a^**
	11	0.04**^a^**	0.17**^abc^**	1.98**^a^**	0.42**^f^**	0.34**^f^**	50.72**^ab^**	10.83**^a^**	8.75**^a^**
	12	0.06**^a^**	0.14**^bc^**	1.95**^a^**	0.47**^ef^**	0.37**^ef^**	39.66**^ab^**	9.00**^a^**	6.98**^a^**
	13	0.06**^a^**	0.14**^bc^**	2.08**^a^**	0.44**^ef^**	0.34**^f^**	49.37**^ab^**	9.91**^a^**	7.52**^a^**
	14	0.13**^a^**	0.17**^abc^**	1.94**^a^**	0.47**^ef^**	0.38**^def^**	35.60**^ab^**	8.14**^a^**	6.31**^a^**
	15	0.11**^a^**	0.19**^ab^**	1.55**^a^**	0.57**^cdef^**	0.44**^bcdef^**	21.10**^ab^**	7.02**^a^**	5.21**^a^**
	16	0.17**^a^**	0.11**^bc^**	1.72**^a^**	0.74**^abcde^**	0.57**^bcde^**	19.30**^ab^**	7.37**^a^**	5.43**^a^**
	17	0.03**^a^**	0.17**^abc^**	1.91**^a^**	0.50**^ef^**	0.32**^f^**	81.62**^a^**	19.03**^a^**	12.56**^a^**
	18	0.11**^a^**	0.29**^a^**	1.85**^a^**	0.82**^abc^**	0.82**^a^**	18.9**^ab^**	8.09**^a^**	8.12**^a^**
	19	0.11**^a^**	0.12**^bc^**	1.60**^a^**	0.96**^a^**	0.60**^bcd^**	16.30**^ab^**	10.50**^a^**	6.38**^a^**
	20	0.13**^a^**	0.10**^bc^**	1.6**^a^**	0.79**^abcd^**	0.54**^bcdef^**	12.75**^b^**	6.26**^a^**	4.27**^a^**
*Saline water*	1	0.15**^ab^**	0.13**^ab^**	1.40**^bc^**	0.54**^ab^**	0.41**^ab^**	11.67**^a^**	4.22**^ab^**	3.12**^ab^**
	2	0.06**^b^**	0.14**^ab^**	1.43**^abc^**	0.46**^ab^**	0.38**^b^**	26.30**^a^**	8.19**^a^**	6.65**^ab^**
	3	0.06**^b^**	0.14**^ab^**	1.39**^bc^**	0.46**^ab^**	0.45**^ab^**	26.65**^a^**	7.96**^ab^**	7.99**^a^**
	4	0.29**^ab^**	0.16**^ab^**	1.29**^c^**	0.61**^ab^**	0.59**^ab^**	7.62**^a^**	3.29**^ab^**	3.09**^ab^**
	5	0.20**^ab^**	0.13**^ab^**	1.38**^bc^**	0.77**^a^**	0.69**^a^**	8.24**^a^**	4.39**^ab^**	4.12**^ab^**
	6	0.25**^ab^**	0.05**^b^**	1.95**^ab^**	0.79**^a^**	0.54**^ab^**	11.85**^a^**	4.00**^ab^**	2.71**^ab^**
	7	0.20**^ab^**	0.09**^b^**	1.64**^abc^**	0.66**^ab^**	0.66**^ab^**	9.33**^a^**	3.74**^ab^**	3.64**^ab^**
	8	0.11**^b^**	0.15**^ab^**	1.49**^abc^**	0.43**^b^**	0.42**^ab^**	14.04**^a^**	3.97**^ab^**	3.86**^ab^**
	9	0.15**^ab^**	0.12**^ab^**	1.50**^abc^**	0.66**^ab^**	0.57**^ab^**	11.11**^a^**	5.05**^ab^**	3.35**^ab^**
	10	0.12^−^	0.07^−^	1.46^−^	0.81^−^	0.78^−^	12.08^−^	6.70^−^	6.39^−^
	11	0.16**^ab^**	0.10**^b^**	1.87**^abc^**	0.47**^ab^**	0.41**^ab^**	21.30**^a^**	4.34**^ab^**	2.14**^ab^**
	12	0.47**^a^**	0.10**^b^**	1.43**^abc^**	0.59**^ab^**	0.63**^ab^**	3.28**^a^**	1.34**^b^**	1.39**^b^**
	13	0.20**^ab^**	0.13**^ab^**	1.63**^abc^**	0.48**^ab^**	0.46**^ab^**	10.29**^a^**	2.79**^ab^**	2.61**^ab^**
	14	0.24**^ab^**	0.12**^ab^**	1.65**^abc^**	0.48**^ab^**	0.45**^ab^**	10.74**^a^**	2.86**^ab^**	2.54**^ab^**
	15	0.14**^ab^**	0.24**^a^**	1.55**^abc^**	0.50 **^ab^**	0.52**^ab^**	13.23**^a^**	4.20**^ab^**	4.41**^ab^**
	16	0.15**^ab^**	0.10**^b^**	1.76**^abc^**	0.64 **^ab^**	0.57**^ab^**	14.45**^a^**	5.16**^ab^**	4.55**^ab^**
	17	0.11**^b^**	0.15**^ab^**	2.05**^a^**	0.57**^ab^**	0.54**^ab^**	35.87**^a^**	4.53**^ab^**	7.19**^ab^**
	18	0.37^−^	0.32^−^	1.56^−^	1.23^−^	1.18^−^	4.26^−^	3.34^−^	3.22^−^
	19	0.21**^ab^**	0.12**^ab^**	1.63**^abc^**	0.77**^a^**	0.67**^ab^**	8.45**^a^**	3.97**^ab^**	3.39**^ab^**
	20	0.26**^ab^**	0.08**^b^**	1.56**^abc^**	0.67**^ab^**	0.59**^ab^**	6.19**^a^**	2.63**^ab^**	2.28**^ab^**

**Table 3:** Average chlorophyll, anthocyanin and flavonoid contents (arbitrary units) and the nitrogen balance index (NBI), of fully expanded leaves of in quinoa accessions grown for eight weeks under different (fresh water and saline) irrigation treatments. Means exhibiting different letters are significantly different (P < 0.05) by the post-hoc Tukey-b test on independent samples within each treatment (fresh and saline water). Values for accession 18 under saline irrigation conditions are not included in the median separation because of the lack of replications. Genotype numbers as detailed in Table 1.

Treatment	Genotype	Pigments			
		Chlorophyll	Anthocyanins	Flavonoids	NBI

*Fresh water*	1	29.34**^ab^**	0.13**^ab^**	1.44**^bcd^**	20.99**^ab^**
	2	30.84**^ab^**	0.12**^ab^**	1.54**^abc^**	20.19**^ab^**
	3	28.15**^ab^**	0.12**^ab^**	1.48**^abcd^**	19.83**^ab^**
	4	31.75**^a^**	0.11**^b^**	1.61**^ab^**	19.91**^ab^**
	5	28.69**^ab^**	0.12**^ab^**	1.59**^ab^**	18.29**^ab^**
	6	27.67**^ab^**	0.12**^ab^**	1.26**^d^**	22.36**^a^**
	7	28.40**^ab^**	0.12**^ab^**	1.32**^cd^**	24.84**^a^**
	8	29.56**^ab^**	0.13**^ab^**	1.57**^abc^**	19.16**^ab^**
	9	26.71**^ab^**	0.14**^ab^**	1.70**^ab^**	16.11**^ab^**
	10	29.89**^ab^**	0.12**^ab^**	1.57**^abc^**	19.22**^ab^**
	11	25.27**^ab^**	0.15**^a^**	1.74**^a^**	14.70**^ab^**
	12	28.96**^ab^**	0.13**^ab^**	1.61**^ab^**	18.13**^ab^**
	13	28.89**^ab^**	0.13**^ab^**	1.64**^ab^**	18.00**^ab^**
	14	23.66**^b^**	0.15**^a^**	1.66**^ab^**	14.26**^b^**
	15	30.55**^ab^**	0.13**^ab^**	1.69**^ab^**	18.11**^ab^**
	16	26.48**^ab^**	0.13**^ab^**	0.49**^abcd^**	18.24**^ab^**
	17	29.27**^ab^**	0.13**^ab^**	1.66**^ab^**	18.00**^ab^**
	18	29.04**^ab^**	0.12**^ab^**	1.64**^ab^**	18.01**^ab^**
	19	32.63**^a^**	0.13**^ab^**	1.61**^ab^**	20.35**^ab^**
	20	29.70**^ab^**	0.12**^ab^**	1.59**^ab^**	18.83**^ab^**
*Saline water*	1	33.85**^ab^**	0.12**^b^**	1.55**^ab^**	21.98**^abc^**
	2	35.46**^ab^**	0.10**^b^**	1.57**^ab^**	22.76**^ab^**
	3	35.33**^ab^**	0.11**^b^**	1.70**^ab^**	21.15**^abc^**
	4	34.28**^ab^**	0.11**^b^**	1.61**^ab^**	21.66**^abc^**
	5	30.72**^ab^**	0.13**^ab^**	1.84**^a^**	16.93**^bc^**
	6	34.44**^ab^**	0.11**^b^**	1.41**^b^**	24.82**^a^**
	7	34.29**^ab^**	0.11**^b^**	1.40**^b^**	25.08**^a^**
	8	31.25**^ab^**	0.13**^ab^**	1.80**^a^**	17.64**^bc^**
	9	35.43**^ab^**	0.11**^b^**	1.81**^a^**	19.78**^abc^**
	10	35.68**^ab^**	0.11**^b^**	1.59**^ab^**	22.42**^abc^**
	11	29.31**^ab^**	0.13**^ab^**	1.78**^a^**	16.63**^bc^**
	12	27.40**^b^**	0.15**^a^**	1.76**^a^**	15.92**^c^**
	13	29.88**^ab^**	0.14**^ab^**	1.76**^a^**	17.24**^bc^**
	14	27.90**^b^**	0.14**^ab^**	1.76**^a^**	16.04**^bc^**
	15	32.50**^ab^**	0.12**^ab^**	1.76**^a^**	18.55**^abc^**
	16	31.83**^ab^**	0.12**^b^**	1.62**^ab^**	19.83**^abc^**
	17	34.94**^ab^**	0.11**^b^**	1.62**^ab^**	22.29**^abc^**
	18	19.04^−^	0.27^−^	1.54^−^	12.90^−^
	19	36.95**^a^**	0.13**^ab^**	1.78**^a^**	21.10**^abc^**
	20	35.38**^ab^**	0.11**^b^**	1.64**^ab^**	22.33**^abc^**

**Table 4:** Average carbon and nitrogen concentrations on a dry matter basis, and carbon (δ^13^C) and nitrogen (δ^15^N) isotope composition in the dry matter and soluble fraction of fully expanded leaves of quinoa accessions grown for eight weeks under different (fresh water and saline) irrigation treatments. Means exhibiting different letters are significantly different (P < 0.05) by the post-hoc Tukey-b test on independent samples within each treatment (control and salinity). Values for accession 18 under saline irrigation conditions are not included in the median separation because of the lack of replications. Genotype numbers as detailed in Table 1.

	Elemental analysis and stable isotopes (dry matter)					Stable isotopes (soluble fraction)	
Treatment	Genotype	C (%)	N (%)	δ^13^C (‰)	δ^15^N (‰)	δ^13^C (‰)	δ^15^N (‰)

*Fresh water*	1	37.01**^ab^**	3.57**^ab^**	-29.27**^a^**	14.20**^a^**	-30.74**^a^**	10.06**^a^**
	2	38.36**^a^**	3.68**^ab^**	-29.39**^a^**	13.04**^a^**	-30.80**^a^**	8.75**^a^**
	3	38.00**^a^**	3.41**^ab^**	-29.99**^a^**	13.59**^a^**	-31.20**^a^**	10.39**^a^**
	4	38.01**^a^**	3.86**^ab^**	-29.61**^a^**	11.99**^a^**	-30.66**^a^**	10.86**^a^**
	5	35.91**^ab^**	3.13**^ab^**	-29.52**^a^**	13.37**^a^**	-32.15**^a^**	8.72**^a^**
	6	35.61**^ab^**	3.10**^ab^**	-29.67**^a^**	11.52**^a^**	-30.67**^a^**	7.35**^a^**
	7	35.74**^ab^**	3.70**^ab^**	-29.12**^a^**	13.61**^a^**	-30.89**^a^**	10.52**^a^**
	8	37.49**^a^**	3.23**^ab^**	-30.04**^a^**	13.58**^a^**	-30.90**^a^**	11.54**^a^**
	9	36.39**^ab^**	3.27**^ab^**	-29.48**^a^**	13.25**^a^**	-32.12**^a^**	10.36**^a^**
	10	35.76**^ab^**	4.19**^a^**	-28.70**^a^**	14.42**^a^**	-30.08**^a^**	12.67**^a^**
	11	37.09**^ab^**	3.14**^ab^**	-29.01**^a^**	15.02**^a^**	-31.01**^a^**	9.18**^a^**
	12	37.76**^a^**	3.81**^ab^**	-29.25**^a^**	15.08**^a^**	-30.37**^a^**	8.10**^a^**
	13	37.58**^a^**	3.77**^ab^**	-28.99**^a^**	14.74**^a^**	-30.62**^a^**	13.36**^a^**
	14	35.55**^ab^**	2.79**^b^**	-28.61**^a^**	12.52**^a^**	-30.92**^a^**	10.30**^a^**
	15	37.06**^ab^**	3.68**^ab^**	-29.50**^a^**	15.98**^a^**	-31.19**^a^**	11.87**^a^**
	16	35.61**^ab^**	3.44**^ab^**	-29.44**^a^**	13.67**^a^**	-30.78**^a^**	9.88**^a^**
	17	37.50**^a^**	3.45**^ab^**	-28.64**^a^**	15.51**^a^**	-30.83**^a^**	9.49**^a^**
	18	32.76**^b^**	2.89**^b^**	-29.11**^a^**	11.46**^a^**	-30.74**^a^**	11.30**^a^**
	19	35.65**^ab^**	3.56**^ab^**	-28.70**^a^**	15.04**^a^**	-30.89**^a^**	11.92**^a^**
	20	36.04**^ab^**	3.37**^ab^**	-28.77**^a^**	13.56**^a^**	-31.33**^a^**	7.50**^a^**
*Saline water*	1	35.64**^a^**	3.20**^a^**	-28.98**^a^**	11.00**^a^**	-31.10**^a^**	7.64**^a^**
	2	37.14**^a^**	3.48**^a^**	-29.18**^a^**	11.58**^a^**	-30.49**^a^**	10.07**^a^**
	3	37.20**^a^**	3.24**^a^**	-28.93**^a^**	11.89**^a^**	-30.81**^a^**	8.20**^a^**
	4	35.38**^a^**	3.49**^a^**	-29.01**^a^**	9.43**^a^**	-30.88**^a^**	5.86**^a^**
	5	33.51**^a^**	2.56**^a^**	-29.37**^a^**	8.67**^a^**	-31.28**^a^**	3.69**^a^**
	6	33.48**^a^**	3.13**^a^**	-29.25**^a^**	7.50**^a^**	-30.66**^a^**	7.09**^a^**
	7	34.33**^a^**	3.56**^a^**	-28.68**^a^**	11.37**^a^**	-30.65**^a^**	8.85**^a^**
	8	36.42**^a^**	2.81**^a^**	-29.72**^a^**	11.25**^a^**	-31.49**^a^**	8.27**^a^**
	9	34.27**^a^**	2.97**^a^**	-28.68**^a^**	8.46**^a^**	-31.38**^a^**	6.40**^a^**
	10	34.66^−^	3.98^−^	-28.54^−^	14.9^−^	-31.75^−^	13.78^−^
	11	32.64**^a^**	2.93**^a^**	-28.58**^a^**	11.92**^a^**	-30.18**^a^**	8.62**^a^**
	12	34.53**^a^**	3.43**^a^**	-28.71**^a^**	11.09**^a^**	-30.84**^a^**	8.81**^a^**
	13	36.37**^a^**	3.55**^a^**	-28.57**^a^**	12.71**^a^**	-30.40**^a^**	9.49**^a^**
	14	34.68**^a^**	2.96**^a^**	-28.74**^a^**	11.34**^a^**	-30.63**^a^**	6.73**^a^**
	15	34.84**^a^**	3.52**^a^**	-29.26**^a^**	14.61**^a^**	-30.86**^a^**	11.19**^a^**
	16	34.61**^a^**	3.70**^a^**	-28.95**^a^**	15.09**^a^**	-31.33**^a^**	10.23**^a^**
	17	35.66**^a^**	3.69**^a^**	-28.57**^a^**	12.68**^a^**	-30.08**^a^**	9.94**^a^**
	18	28.23^−^	2.19^−^	-26.66^−^	9.19^−^	-29.58^−^	7.53^−^
	19	33.58**^a^**	3.19**^a^**	-28.23**^a^**	10.42**^a^**	-30.57**^a^**	8.86**^a^**
	20	34.53**^a^**	3.31**^a^**	-28.63**^a^**	10.76**^a^**	-31.15**^a^**	7.82**^a^**

## Experimental Design, Materials, and Methods

Two field experiments were planted on November 19^th^, 2016. Quinoa seeds were sown by hand following a randomized complete block design with three replicates per genotype. Plot size was 2 × 2 meters, with a plant-to-plant distance of 25 cm and 50 cm between rows, totaling 45 plants per plot (5 × 9). During the two first weeks, both trials were supplied with fresh water drip-irrigation (1 dS m^−1^) to avoid hindering germination. Then, two different treatments were imposed for the rest of the growing period to a) irrigation with fresh water and b) irrigation with saline water (15 dS m-1).

Eight weeks after treatments application, 10 fully expanded leaves were assessed randomly from the central rows of each plot in both trials, using a leaf pigment meter (Dualex). The same leaves were collected, dried, ground to a fine powder and analyzed for ion concentration determination using an Inductively Coupled Plasma Emission Spectrometer (ICPES), and stable isotope composition and elemental analysis determination, using an elemental analyzer coupled with an isotope ratio mass spectrometer (EA-IRMS).

At physiological maturity, yield components were assessed as described previously in Hussain et al. [3]: 5 plants were selected from the central rows. Height was measured from the ground to the top of inflorescence on the main stem. Similarly, the number of branches was recorded at different node positions of the main stem including basal branches. The number of inflorescences per plant was counted, and the length of 3 random inflorescences was averaged. Biomass and seed yield were assessed by manually harvesting the 5 plants from the middle row of each plot.

Average, minimum and maximum temperature and precipitation data were obtained from the meteorological station of the International Center for Biosaline Agriculture (ICBA)

Raw data were analyzed using the statistical package SPSS (SPSS Inc.), using a multivariate analysis coupled with the post hoc test (Tukey-b) to assist differences between genotypes within each treatment.

Graphs were created using the SigmaPlot program 10.0 (SPSS Inc.).

## Declaration of Competing Interest

The authors declare that they have no known competing financial interests or personal relationships that have, or could be perceived to have, influenced the work reported in this article.

## Appendix Supplementary materials

Image, application 1
