# Supplementary material for: Agronomical and analytical trait data assessed in a set of quinoa genotypes growing in the UAE under different irrigation salinity conditions
Source: Data Brief. 2020 May 30;31:105758. doi: 10.1016/j.dib.2020.105758 (PMC7300274; doi:10.1016/j.dib.2020.105758)
Supplement: Supplementary file 1 [file mmc1.pdf]

Data for: agronomical and analytical traits assessed in a quinoa set of genotypes growing in the UAE under different irrigation salinity conditions

Raw data Table 1. Raw data of yield components (seed yield, biomass, plant height, branches per plant, inflorescences per plant, inflorescence length) in the 3 replicates of quinoa accessions grown under fresh water and saline irrigation treatments.

		Yield components					
Treatment	Genotype	Seed yield (g m^{-2})	Biomass (g m^{-2})	Plant height (cm)	Branches $plant^{-1}$	Inflorescences $plant^{-1}$	Inflorescence length (cm)
Fresh water	1	444,3	1440,0	90,8	8,2	6,6	24,7
Fresh water	1	340,0	2175,0	94,7	8,8	8,6	25,5
Fresh water	1	730,2	3060,0	122,2	7,8	7,6	30,5
Fresh water	2	425,7	1860,0	127,2	7,2	5,8	42,7
Fresh water	2	407,7	1800,0	117,9	7,8	7,8	35,5
Fresh water	2	544,5	2220,0	126,3	8,0	7,0	41,5
Fresh water	3	331,8	1560,0	154,8	3,6	3,2	56,5
Fresh water	3	498,9	3900,0	137,2	4,6	4,2	46,7
Fresh water	3	345,9	2580,0	142,5	5,6	4,6	49,3
Fresh water	4	296,7	1380,0	109,0	7,2	5,8	32,8
Fresh water	4	373,2	1860,0	126,5	7,4	7,0	37,6
Fresh water	4	532,2	2940,0	130,8	6,2	5,0	45,0
Fresh water	5	357,9	1980,0	83,1	5,0	4,6	26,0
Fresh water	5	265,0	1100,0	86,2	6,2	6,2	29,0
Fresh water	5	268,0	1200,0	95,2	6,8	5,8	28,7
Fresh water	6	411,3	3120,0	98,7	8,2	6,2	35,7
Fresh water	6	461,1	2400,0	85,6	9,4	8,2	31,2
Fresh water	6	412,8	1860,0	82,4	9,8	9,2	32,3
Fresh water	7	418,8	1560,0	95,1	5,4	5,2	31,9
Fresh water	7	486,0	2280,0	102,6	8,4	6,8	37,5
Fresh water	7	625,2	2160,0	104,0	8,0	7,6	35,7
Fresh water	8	407,7	1740,0	113,6	6,8	3,8	32,4
Fresh water	8	573,9	1980,0	112,7	9,2	5,6	40,3
Fresh water	8	651,3	2100,0	134,6	7,0	6,2	47,7
Fresh water	9	327,9	1200,0	112,9	6,0	4,6	30,6
Fresh water	9	537,0	1860,0	112,1	9,8	4,6	31,8
Fresh water	9	339,0	1140,0	119,6	6,6	5,2	38,9
Fresh water	10	59,7	780,0	54,7	6,6	6,4	17,5
Fresh water	10	-	-	43,9	6,8	6,6	17,2
Fresh water	10	24,6	600,0	42,1	8,0	7,6	19,2
Fresh water	11	491,7	2820,0	102,8	6,8	6,4	28,5
Fresh water	11	444,6	2580,0	113,0	8,8	5,8	38,1
Fresh water	11	270,9	3360,0	119,3	8,6	6,8	37,7
Fresh water	12	547,8	3000,0	113,4	7,4	6,4	38,0
Fresh water	12	370,5	2340,0	105,4	10,0	7,8	35,4
Fresh water	12	402,6	3900,0	134,8	15,8	13,0	43,8
Fresh water	13	496,2	3540,0	105,5	9,6	8,8	36,1
Fresh water	13	687,9	3840,0	109,1	8,0	7,2	37,7
Fresh water	13	446,7	2940,0	108,9	9,2	8,4	33,5
Fresh water	14	347,1	2100,0	105,2	7,8	5,8	37,2
Fresh water	14	236,7	1380,0	108,2	9,0	8,6	28,0
Fresh water	14	506,1	2760,0	116,7	8,0	6,6	34,9
Fresh water	15	435,9	2520,0	72,9	8,8	7,4	25,4
Fresh water	15	243,0	2400,0	56,0	6,2	6,2	21,8

Fresh water	15	291,3	1620,0	56,6	8,4	6,6	24,7
Fresh water	16	103,2	1800,0	61,6	6,6	5,8	24,1
Fresh water	16	56,0	1300,0	47,4	6,6	6,4	21,6
Fresh water	16	95,0	1350,0	52,5	5,8	5,4	20,9
Fresh water	17	729,9	2940,0	111,8	9,8	8,8	31,9
Fresh water	17	770,0	3075,0	106,7	6,8	6,6	30,5
Fresh water	17	397,2	2040,0	115,6	8,6	6,6	39,4
Fresh water	18	44,1	540,0	29,1	7,2	7,2	12,6
Fresh water	18	54,6	438,0	23,9	5,2	4,6	10,4
Fresh water	18	53,7	414,0	23,1	4,8	4,6	8,5
Fresh water	19	320,4	2040,0	101,3	7,0	7,0	30,9
Fresh water	19	676,5	2520,0	98,1	7,0	7,0	30,6
Fresh water	19	512,1	1800,0	114,1	8,8	7,4	37,4
Fresh water	20	294,0	960,0	83,0	5,2	4,8	30,7
Fresh water	20	369,3	2025,0	77,2	11,4	9,6	27,3
Fresh water	20	399,9	1200,0	71,5	9,6	9,2	30,1
Saline water	1	375,3	1800,0	91,4	5,8	4,8	28,0
Saline water	1	395,8	1100,0	74,9	7,0	6,8	23,1
Saline water	1	387,6	1560,0	87,5	7,4	6,6	25,5
Saline water	2	189,3	1380,0	74,4	7,0	6,6	24,7
Saline water	2	218,7	1200,0	92,2	5,6	5,0	29,1
Saline water	2	153,9	840,0	90,5	7,6	5,6	30,2
Saline water	3	166,2	2040,0	110,4	6,2	5,2	38,2
Saline water	3	197,7	1440,0	110,3	4,8	4,4	41,0
Saline water	3	300,6	2340,0	124,5	7,2	6,4	39,4
Saline water	4	169,2	1320,0	120,5	10,2	8,4	39,1
Saline water	4	401,1	2580,0	100,6	9,0	8,2	34,0
Saline water	4	176,7	1200,0	98,4	11,8	9,8	33,9
Saline water	5	263,3	2400,0	84,4	6,2	5,2	30,8
Saline water	5	180,6	900,0	83,2	6,8	5,8	34,4
Saline water	5	206,4	930,0	68,0	7,0	6,4	26,6
Saline water	6	489,3	1920,0	86,0	7,2	7,0	31,7
Saline water	6	477,9	1860,0	80,7	5,0	5,4	27,2
Saline water	6	360,0	1050,0	76,0	4,0	3,8	27,7
Saline water	7	244,2	1080,0	69,8	4,0	3,8	29,5
Saline water	7	330,9	1680,0	63,2	5,0	4,8	24,4
Saline water	7	285,6	1140,0	64,0	5,4	5,2	28,2
Saline water	8	357,3	1620,0	84,8	6,6	6,2	31,4
Saline water	8	318,9	1560,0	81,2	5,2	5,0	30,8
Saline water	8	462,3	1680,0	100,8	7,2	6,2	28,9
Saline water	9	407,4	2100,0	100,9	6,4	6,0	34,2
Saline water	9	259,8	1020,0	78,2	6,2	5,8	32,3
Saline water	9	202,2	1020,0	75,9	5,6	5,0	27,7
Saline water	10	-	-	5,7	0,6	0,6	3,0
Saline water	10	-	-	57,4	5,2	5,0	26,0
Saline water	10	-	-	-	-	-	-
Saline water	11	323,1	3480,0	85,9	9,6	9,6	38,0
Saline water	11	478,5	3060,0	89,8	7,0	7,0	28,3
Saline water	11	446,7	3900,0	98,4	6,0	6,0	35,1
Saline water	12	119,5	2300,0	35,0	7,4	7,0	17,4
Saline water	12	113,4	2220,0	54,7	6,8	6,4	27,9
Saline water	12	89,4	600,0	54,1	5,2	5,0	28,4
Saline water	13	191,7	2700,0	72,5	6,4	6,4	31,7
Saline water	13	393,3	2700,0	91,1	5,4	5,4	35,8
Saline water	13	260,4	2580,0	88,1	6,6	6,6	30,0
Saline water	14	409,8	2940,0	85,7	6,6	6,2	38,3
Saline water	14	309,9	1440,0	75,3	7,8	7,8	31,0
Saline water	14	420,6	2220,0	81,8	6,0	5,6	33,8

Saline water	15	212,0	1800,0	59,0	3,2	3,0	21,9
Saline water	15	202,9	1125,0	54,6	6,0	5,0	20,3
Saline water	15	180,0	800,0	44,5	6,6	6,0	23,2
Saline water	16	206,5	2000,0	49,4	7,4	5,6	19,1
Saline water	16	213,0	2400,0	40,7	6,4	5,4	18,9
Saline water	16	261,0	4500,0	44,9	6,6	5,6	18,7
Saline water	17	404,4	1935,0	97,9	7,4	6,0	31,3
Saline water	17	324,0	1050,0	69,7	5,6	5,4	24,1
Saline water	17	434,1	2160,0	80,4	6,2	5,6	28,2
Saline water	18	39,0	900,0	20,1	2,8	2,8	8,2
Saline water	18	-	-	21,2	2,6	2,6	10,6
Saline water	18	64,5	1800,0	22,3	3,0	2,6	9,9
Saline water	19	385,8	1320,0	67,8	5,4	5,2	25,6
Saline water	19	216,3	960,0	71,6	6,6	6,4	29,3
Saline water	19	238,2	900,0	59,3	6,2	5,8	24,0
Saline water	20	153,0	1500,0	38,3	4,4	4,4	17,5
Saline water	20	186,0	900,0	50,2	4,8	4,8	19,1
Saline water	20	226,8	720,0	43,7	5,6	4,6	21,3

Raw data Table 2. Raw data of ion concentrations (sodium, phosphorus, potassium, calcium and magnesium concentrations and the K^+/Na^+, Ca^{2+}/Na^+ and Mg^{2+}/Na^+) in the 3 replicates of quinoa accessions grown under fresh water and saline irrigation treatments.

		Ion concentration					Ratios		
Treatment	Genotype	Na^+ (mmol. g^{-1})	P (mmol. g^{-1})	K^+ (mmol. g^{-1})	Ca^{2+} (mmol. g^{-1})	Mg^{2+} (mmol. g^{-1})	K^+/Na^+	Ca^{2+}/Na^+	Mg^{2+}/Na^+
Fresh water	1	0,048	0,190	1,478	0,473	0,292	31,05	9,93	6,13
Fresh water	1	0,045	0,130	1,569	0,439	0,281	34,57	9,68	6,19
Fresh water	1	0,071	0,158	1,778	0,524	0,393	24,98	7,36	5,52
Fresh water	2	0,023	0,175	1,663	0,534	0,376	71,78	23,06	16,25
Fresh water	2	0,027	0,141	1,708	0,486	0,411	63,83	18,17	15,34
Fresh water	2	0,028	0,146	1,583	0,459	0,285	56,57	16,41	10,18
Fresh water	3	0,025	0,232	1,619	0,487	0,478	63,63	19,14	18,79
Fresh water	3	0,038	0,169	1,400	0,419	0,426	36,85	11,02	11,21
Fresh water	3	0,043	0,147	1,557	0,552	0,398	36,59	12,96	9,36
Fresh water	4	0,110	0,144	1,253	0,627	0,522	11,44	5,72	4,76
Fresh water	4	0,024	0,237	1,937	0,528	0,397	79,75	21,74	16,34
Fresh water	4	0,037	0,178	1,528	0,623	0,340	41,32	16,84	9,20
Fresh water	5	0,049	0,112	1,609	0,815	0,620	33,10	16,77	12,77
Fresh water	5	0,073	0,054	1,310	0,896	0,622	17,99	12,31	8,54
Fresh water	5	0,082	0,098	1,569	0,821	0,607	19,15	10,02	7,41
Fresh water	6	0,078	0,047	1,997	0,677	0,361	25,64	8,70	4,63
Fresh water	6	0,156	0,053	2,167	0,792	0,462	13,91	5,09	2,97
Fresh water	6	0,181	0,048	1,916	0,862	0,423	10,60	4,77	2,34
Fresh water	7	0,090	0,092	1,814	0,742	0,677	20,19	8,26	7,54
Fresh water	7	0,058	0,108	2,154	0,599	0,543	37,42	10,41	9,44
Fresh water	7	0,098	0,121	1,961	0,663	0,620	20,09	6,80	6,35
Fresh water	8	0,045	0,178	1,369	0,524	0,406	30,51	11,68	9,05
Fresh water	8	0,068	0,154	1,475	0,498	0,326	21,66	7,31	4,79
Fresh water	8	0,040	0,207	1,463	0,566	0,342	36,35	14,07	8,50
Fresh water	9	0,028	0,197	1,537	0,645	0,521	55,83	23,44	18,94
Fresh water	9	0,082	0,160	1,397	0,895	0,542	17,00	10,89	6,59
Fresh water	9	0,045	0,161	1,416	0,883	0,493	31,15	19,42	10,83
Fresh water	10	0,113	0,096	2,032	0,760	0,735	17,92	6,70	6,48
Fresh water	10	0,057	0,096	2,008	0,729	0,741	35,16	12,76	12,97
Fresh water	10	0,075	0,090	1,822	0,604	0,559	24,25	8,03	7,44
Fresh water	11	0,028	0,186	1,913	0,398	0,348	68,31	14,21	12,44
Fresh water	11	0,037	0,150	2,042	0,460	0,326	54,80	12,33	8,74
Fresh water	11	0,068	0,187	1,980	0,404	0,345	29,07	5,93	5,06
Fresh water	12	0,088	0,102	1,913	0,581	0,493	21,72	6,59	5,60
Fresh water	12	0,034	0,190	2,083	0,448	0,318	61,93	13,32	9,46
Fresh water	12	0,052	0,142	1,852	0,372	0,307	35,35	7,10	5,87
Fresh water	13	0,064	0,141	2,164	0,479	0,377	33,85	7,49	5,90
Fresh water	13	0,025	0,130	2,300	0,429	0,322	93,59	17,48	13,10
Fresh water	13	0,086	0,160	1,777	0,409	0,307	20,67	4,75	3,57
Fresh water	14	0,041	0,168	1,774	0,444	0,364	43,22	10,83	8,87
Fresh water	14	0,304	0,158	1,844	0,502	0,454	6,07	1,65	1,49
Fresh water	14	0,038	0,173	2,203	0,457	0,328	57,50	11,93	8,55
Fresh water	15	0,082	0,104	1,682	0,606	0,501	20,62	7,43	6,14
Fresh water	15	0,218	0,233	1,459	0,637	0,522	6,69	2,92	2,39
Fresh water	15	0,042	0,233	1,523	0,453	0,300	36,01	10,70	7,09
Fresh water	16	0,053	0,066	2,420	0,851	0,602	45,53	16,02	11,33
Fresh water	16	0,203	0,129	1,520	0,623	0,516	7,49	3,07	2,54
Fresh water	16	0,247	0,122	1,209	0,748	0,598	4,89	3,03	2,42
Fresh water	17	0,067	0,143	1,547	0,594	0,362	23,04	8,84	5,40

Fresh water	17	0,021	0,191	2,358	0,475	0,316	112,96	22,77	15,15
Fresh water	17	0,017	0,170	1,822	0,426	0,287	108,87	25,48	17,14
Fresh water	18	0,114	0,178	1,882	0,917	1,015	16,55	8,06	8,93
Fresh water	18	0,139	0,411	1,861	0,892	0,803	13,40	6,43	5,78
Fresh water	18	0,068	0,270	1,816	0,663	0,654	26,83	9,79	9,66
Fresh water	19	0,068	0,086	1,459	1,209	0,683	21,48	17,80	10,05
Fresh water	19	0,138	0,135	1,484	0,786	0,573	10,73	5,68	4,14
Fresh water	19	0,111	0,131	1,848	0,887	0,547	16,70	8,01	4,94
Fresh water	20	0,112	0,061	1,746	0,866	0,558	15,55	7,72	4,97
Fresh water	20	0,114	0,124	1,468	0,726	0,510	12,92	6,40	4,49
Fresh water	20	0,165	0,104	1,613	0,772	0,555	9,77	4,67	3,36
Saline water	1	0,112	0,129	1,475	0,631	0,452	13,23	5,66	4,05
Saline water	1	0,231	0,054	1,161	0,568	0,463	5,03	2,46	2,01
Saline water	1	0,094	0,217	1,570	0,425	0,310	16,75	4,53	3,30
Saline water	2	0,086	0,092	1,370	0,533	0,475	15,86	6,17	5,50
Saline water	2	0,052	0,149	1,444	0,433	0,342	27,67	8,29	6,54
Saline water	2	0,042	0,163	1,471	0,420	0,329	35,38	10,09	7,90
Saline water	3	0,106	0,171	1,262	0,617	0,527	11,94	5,83	4,99
Saline water	3	0,051	0,110	1,358	0,397	0,399	26,75	7,82	7,85
Saline water	3	0,037	0,149	1,535	0,381	0,414	41,26	10,23	11,12
Saline water	4	0,090	0,151	1,329	0,510	0,469	14,83	5,69	5,23
Saline water	4	0,212	0,179	1,188	0,620	0,581	5,61	2,93	2,74
Saline water	4	0,563	0,137	1,358	0,711	0,723	2,41	1,26	1,28
Saline water	5	0,304	0,090	1,295	0,868	0,672	4,26	2,85	2,21
Saline water	5	0,121	0,108	1,382	0,652	0,673	11,46	5,41	5,58
Saline water	5	0,162	0,179	1,457	0,794	0,740	9,02	4,91	4,58
Saline water	6	0,100	0,047	2,446	0,669	0,456	24,56	6,72	4,58
Saline water	6	0,270	0,049	2,032	0,822	0,529	7,52	3,04	1,96
Saline water	6	0,395	0,039	1,363	0,885	0,632	3,45	2,24	1,60
Saline water	7	0,241	0,105	1,537	0,596	0,714	6,38	2,48	2,97
Saline water	7	0,238	0,066	1,590	0,703	0,645	6,69	2,96	2,71
Saline water	7	0,120	0,093	1,790	0,695	0,630	14,92	5,79	5,25
Saline water	8	0,151	0,105	1,345	0,446	0,476	8,88	2,95	3,15
Saline water	8	0,112	0,173	1,495	0,401	0,400	13,36	3,58	3,57
Saline water	8	0,082	0,170	1,621	0,438	0,397	19,87	5,37	4,86
Saline water	9	0,111	0,112	1,551	0,590	0,548	13,93	5,29	4,92
Saline water	9	0,108	0,113	1,363	0,773	0,625	12,61	7,15	5,78
Saline water	9	0,231	0,124	1,574	0,627	0,542	6,80	2,71	2,34
Saline water	10	-	-	-	-	-	-	-	-
Saline water	10	0,121	0,073	1,465	0,813	0,775	12,08	6,70	6,39
Saline water	10	-	-	-	-	-	-	-	-
Saline water	11	0,189	0,103	1,784	0,485	0,434	9,45	2,57	2,30
Saline water	11	0,243	0,105	1,626	0,544	0,409	6,68	2,24	1,68
Saline water	11	0,046	0,096	2,186	0,376	0,387	47,76	8,22	8,45
Saline water	12	0,618	0,048	1,290	0,565	0,697	2,09	0,92	1,13
Saline water	12	0,338	0,173	1,490	0,604	0,507	4,41	1,79	1,50
Saline water	12	0,452	0,094	1,512	0,588	0,698	3,34	1,30	1,54
Saline water	13	0,333	0,101	1,501	0,568	0,589	4,50	1,70	1,77
Saline water	13	0,154	0,095	1,589	0,450	0,362	10,32	2,92	2,35
Saline water	13	0,113	0,186	1,812	0,422	0,420	16,04	3,73	3,72
Saline water	14	0,113	0,124	1,828	0,454	0,369	16,23	4,03	3,28
Saline water	14	0,487	0,080	1,342	0,517	0,531	2,76	1,06	1,09
Saline water	14	0,135	0,156	1,780	0,471	0,438	13,22	3,50	3,25
Saline water	15	0,185	0,193	1,688	0,534	0,576	9,14	2,89	3,12
Saline water	15	0,157	0,268	1,501	0,522	0,486	9,55	3,32	3,09
Saline water	15	0,070	0,252	1,464	0,445	0,489	21,01	6,38	7,02
Saline water	16	0,237	0,055	1,619	0,811	0,702	6,82	3,42	2,96
Saline water	16	0,075	0,069	1,615	0,644	0,549	21,56	8,59	7,33

Saline water	16	0,136	0,180	2,038	0,471	0,458	14,97	3,46	3,36
Saline water	17	0,032	0,180	2,320	0,412	0,394	72,30	12,85	12,27
Saline water	17	0,229	0,113	1,920	0,883	0,827	8,39	3,86	3,61
Saline water	17	0,071	0,144	1,915	0,419	0,406	26,92	5,88	5,70
Saline water	18	0,367	0,321	1,565	1,228	1,183	4,26	3,34	3,22
Saline water	18	-	-	-	-	-	-	-	-
Saline water	18	-	-	-	-	-	-	-	-
Saline water	19	0,132	0,078	1,653	0,759	0,639	12,54	5,76	4,85
Saline water	19	0,234	0,158	1,714	0,798	0,616	7,31	3,40	2,63
Saline water	19	0,277	0,132	1,525	0,763	0,748	5,50	2,75	2,70
Saline water	20	0,319	0,043	1,308	0,617	0,553	4,10	1,94	1,73
Saline water	20	0,246	0,107	1,649	0,808	0,682	6,70	3,28	2,77
Saline water	20	0,222	0,081	1,730	0,593	0,522	7,78	2,67	2,35

Raw data Table 3. Raw data of leaf pigments (chlorophyll, anthocyanin and flavonoid contents) in the 3 replicates of quinoa accessions grown under fresh water and saline irrigation treatments.

		Leaf pigments			
Treatment	Genotype	Chlorophylls	Anthocyanins	Flavonoids	NBI
Fresh water	1	27,84	1,48	0,14	19,21
Fresh water	1	29,68	1,53	0,12	19,74
Fresh water	1	30,50	1,30	0,12	24,01
Fresh water	2	30,81	1,54	0,12	20,47
Fresh water	2	29,51	1,53	0,12	19,33
Fresh water	2	32,20	1,56	0,11	20,79
Fresh water	3	26,84	1,63	0,13	16,74
Fresh water	3	30,89	1,39	0,11	23,60
Fresh water	3	26,73	1,42	0,12	19,14
Fresh water	4	33,75	1,53	0,10	22,14
Fresh water	4	30,50	1,70	0,12	18,10
Fresh water	4	30,99	1,60	0,11	19,48
Fresh water	5	33,24	1,54	0,10	21,89
Fresh water	5	28,39	1,69	0,12	16,90
Fresh water	5	24,42	1,54	0,14	16,11
Fresh water	6	27,45	1,26	0,12	21,90
Fresh water	6	25,82	1,24	0,12	21,30
Fresh water	6	29,73	1,27	0,11	23,88
Fresh water	7	28,13	1,29	0,12	22,10
Fresh water	7	28,11	1,37	0,12	20,81
Fresh water	7	28,95	1,30	0,12	22,60
Fresh water	8	28,98	1,70	0,14	17,24
Fresh water	8	30,26	1,58	0,13	19,41
Fresh water	8	29,44	1,42	0,12	20,84
Fresh water	9	29,53	1,74	0,13	17,60
Fresh water	9	24,78	1,71	0,14	14,54
Fresh water	9	25,83	1,65	0,13	16,21
Fresh water	10	30,81	1,54	0,12	20,47
Fresh water	10	27,88	1,59	0,13	17,72
Fresh water	10	30,99	1,60	0,11	19,48
Fresh water	11	23,26	1,80	0,16	12,96
Fresh water	11	26,31	1,73	0,14	15,29
Fresh water	11	26,26	1,68	0,13	15,86
Fresh water	12	29,99	1,55	0,12	19,45
Fresh water	12	25,97	1,62	0,13	16,15
Fresh water	12	30,92	1,66	0,12	18,79
Fresh water	13	26,78	1,70	0,14	16,07
Fresh water	13	30,49	1,61	0,11	19,40
Fresh water	13	29,39	1,61	0,13	18,54
Fresh water	14	23,79	1,77	0,15	13,43
Fresh water	14	17,75	1,58	0,19	11,25
Fresh water	14	29,42	1,63	0,13	18,10
Fresh water	15	32,76	1,69	0,11	19,54
Fresh water	15	26,78	1,66	0,15	16,26
Fresh water	15	32,10	1,74	0,13	18,52
Fresh water	16	26,77	1,25	0,12	21,67
Fresh water	16	27,88	1,59	0,13	17,72
Fresh water	16	24,80	1,66	0,14	15,34
Fresh water	17	32,27	1,50	0,11	21,88
Fresh water	17	26,64	1,78	0,14	15,15
Fresh water	17	28,88	1,71	0,13	16,97
Fresh water	18	26,84	1,63	0,13	16,74

Fresh water	18	29,38	1,61	0,14	18,51
Fresh water	18	30,92	1,66	0,12	18,79
Fresh water	19	35,16	1,68	0,12	21,02
Fresh water	19	29,38	1,61	0,14	18,51
Fresh water	19	33,35	1,56	0,14	21,51
Fresh water	20	28,93	1,60	0,12	18,21
Fresh water	20	29,49	1,57	0,12	19,01
Fresh water	20	30,68	1,61	0,11	19,28
Saline water	1	35,44	1,53	0,11	23,25
Saline water	1	35,56	1,56	0,11	23,19
Saline water	1	30,57	1,57	0,12	19,50
Saline water	2	33,13	1,50	0,12	22,46
Saline water	2	36,11	1,57	0,10	23,14
Saline water	2	37,15	1,65	0,10	22,68
Saline water	3	35,06	1,54	0,10	23,23
Saline water	3	36,71	1,77	0,11	20,98
Saline water	3	34,22	1,79	0,11	19,23
Saline water	4	36,62	1,54	0,10	24,45
Saline water	4	34,30	1,70	0,11	20,39
Saline water	4	31,93	1,59	0,12	20,13
Saline water	5	33,73	1,81	0,13	18,79
Saline water	5	27,75	1,88	0,14	15,04
Saline water	5	30,68	1,83	0,13	16,97
Saline water	6	38,32	1,38	0,09	28,02
Saline water	6	31,63	1,32	0,11	24,14
Saline water	6	33,36	1,52	0,12	22,30
Saline water	7	31,56	1,33	0,13	24,57
Saline water	7	35,30	1,37	0,11	26,36
Saline water	7	36,00	1,50	0,10	24,30
Saline water	8	30,30	1,81	0,14	16,97
Saline water	8	32,48	1,76	0,12	18,65
Saline water	8	30,99	1,84	0,14	17,30
Saline water	9	31,40	1,82	0,13	17,25
Saline water	9	38,81	1,84	0,10	21,38
Saline water	9	36,08	1,75	0,11	20,72
Saline water	10	-	-	-	-
Saline water	10	39,42	1,60	0,11	24,70
Saline water	10	31,93	1,59	0,12	20,13
Saline water	11	26,88	1,85	0,15	14,58
Saline water	11	30,97	1,73	0,12	18,07
Saline water	11	30,09	1,75	0,13	17,23
Saline water	12	26,33	1,59	0,16	16,90
Saline water	12	26,89	1,96	0,16	13,74
Saline water	12	28,99	1,71	0,15	17,14
Saline water	13	33,53	1,69	0,13	19,95
Saline water	13	27,71	1,71	0,13	16,53
Saline water	13	28,41	1,88	0,15	15,25
Saline water	14	29,51	1,76	0,13	17,03
Saline water	14	27,28	1,68	0,14	16,30
Saline water	14	26,90	1,85	0,15	14,78
Saline water	15	30,11	1,71	0,13	17,61
Saline water	15	29,32	1,75	0,13	16,83
Saline water	15	38,07	1,80	0,11	21,21
Saline water	16	27,51	1,45	0,13	19,31
Saline water	16	36,50	1,76	0,12	20,93
Saline water	16	31,48	1,64	0,11	19,25
Saline water	17	33,22	1,31	0,11	25,81
Saline water	17	38,53	1,72	0,12	22,84

Saline water	17	33,07	1,83	0,11	18,21
Saline water	18	19,04	1,54	0,27	12,90
Saline water	18	-	-	-	-
Saline water	18	-	-	-	-
Saline water	19	39,45	1,66	0,12	24,02
Saline water	19	36,81	1,83	0,13	20,37
Saline water	19	34,59	1,85	0,15	18,92
Saline water	20	33,72	1,61	0,11	21,89
Saline water	20	38,61	1,57	0,10	25,33
Saline water	20	33,81	1,73	0,11	19,78

Raw data Table 4. Raw data of carbon and nitrogen concentrations on a dry matter basis, and carbon ($\delta^{13}C$) and nitrogen ($\delta^{15}N$) isotope composition in the dry matter and soluble fraction in the 3 replicates of quinoa accessions grown under fresh water and saline irrigation treatments.

		Elemental analysis and stable isotopes (dry matter)				Stable isotopes (soluble fraction)	
Treatment	Genotype	N (%)	C (%)	$\delta^{15}N$ (‰)	$\delta^{13}C$ (‰)	$\delta^{15}N$ (‰)	$\delta^{13}C$ (‰)
Fresh water	1	2,4	33,0	11,4	-29,4	8,8	-32,7
Fresh water	1	4,1	39,1	14,6	-28,7	9,1	-29,4
Fresh water	1	4,1	38,9	16,6	-29,7	12,3	-30,2
Fresh water	2	4,0	38,7	15,1	-29,2	11,7	-31,1
Fresh water	2	3,7	36,8	11,2	-29,4	6,6	-30,4
Fresh water	2	3,4	39,6	12,7	-29,6	7,9	-30,9
Fresh water	3	3,1	36,8	12,9	-29,5	15,6	-30,1
Fresh water	3	4,1	38,6	16,5	-30,0	8,1	-31,1
Fresh water	3	2,9	38,5	11,4	-30,4	7,5	-32,3
Fresh water	4	3,9	38,3	12,3	-29,2	10,9	-30,0
Fresh water	4	3,7	36,5	11,7	-29,1	11,2	-29,0
Fresh water	4	4,0	39,3	12,0	-30,5	10,4	-33,0
Fresh water	5	3,7	35,6	16,2	-29,4	10,0	-31,4
Fresh water	5	2,8	35,3	9,4	-29,3	5,7	-32,8
Fresh water	5	2,9	36,7	14,6	-29,9	10,5	-32,3
Fresh water	6	3,0	36,5	12,0	-30,3	7,7	-31,0
Fresh water	6	3,0	34,8	11,7	-29,4	5,9	-31,6
Fresh water	6	3,3	35,5	10,8	-29,4	8,4	-29,4
Fresh water	7	3,6	35,8	11,5	-29,6	9,1	-31,8
Fresh water	7	3,7	35,2	15,1	-28,7	10,3	-29,9
Fresh water	7	3,7	36,3	14,3	-29,1	12,2	-31,0
Fresh water	8	2,9	37,3	13,7	-29,7	13,5	-29,5
Fresh water	8	3,6	38,5	13,1	-29,9	10,2	-31,0
Fresh water	8	3,1	36,7	13,9	-30,5	10,9	-32,3
Fresh water	9	3,2	36,9	14,9	-28,9	11,5	-31,4
Fresh water	9	3,5	35,4	13,0	-29,5	9,6	-32,4
Fresh water	9	3,1	36,9	11,9	-30,0	9,9	-32,6
Fresh water	10	4,3	35,3	14,6	-28,6	13,4	-29,4
Fresh water	10	3,8	34,6	11,7	-28,5	9,1	-30,5
Fresh water	10	4,5	37,3	17,0	-29,0	15,5	-30,4
Fresh water	11	2,6	36,5	12,7	-28,5	5,2	-32,2
Fresh water	11	3,7	36,9	15,4	-28,9	10,7	-30,2
Fresh water	11	3,1	37,8	17,0	-29,6	11,6	-30,6
Fresh water	12	4,2	37,1	16,8	-28,9	4,5	-29,8
Fresh water	12	3,3	36,4	10,0	-29,2	6,0	-30,4
Fresh water	12	4,0	39,8	18,4	-29,7	13,8	-30,9
Fresh water	13	3,6	37,0	15,7	-28,7	17,3	-29,6
Fresh water	13	4,1	36,3	14,0	-28,7	11,7	-32,7
Fresh water	13	3,6	39,4	14,4	-29,6	11,1	-29,5
Fresh water	14	2,5	35,9	13,5	-28,5	9,0	-31,3
Fresh water	14	2,7	35,6	8,7	-29,5	11,6	-32,1
Fresh water	14	3,2	35,1	15,3	-27,9	10,3	-29,3
Fresh water	15	4,0	37,5	14,2	-28,9	10,3	-30,2
Fresh water	15	3,4	34,9	15,7	-29,6	12,8	-30,8
Fresh water	15	3,7	38,7	18,0	-30,1	12,5	-32,6
Fresh water	16	3,5	33,5	10,6	-29,4	6,4	-30,1
Fresh water	16	3,1	36,1	13,7	-29,4	9,3	-31,0
Fresh water	16	3,7	37,3	16,8	-29,6	13,9	-31,2
Fresh water	17	3,2	36,8	14,7	-29,4	6,8	-32,0
Fresh water	17	3,6	36,6	15,8	-28,1	11,8	-31,3

Fresh water	17	3,6	39,1	16,0	-28,5	9,8	-29,2
Fresh water	18	3,3	32,1	11,0	-29,1	13,2	-30,5
Fresh water	18	2,8	31,3	11,1	-29,1	9,6	-31,1
Fresh water	18	2,6	34,9	12,3	-29,1	11,1	-30,6
Fresh water	19	3,7	35,7	14,9	-28,1	12,7	-30,6
Fresh water	19	3,2	35,4	16,5	-28,9	13,0	-30,9
Fresh water	19	3,7	35,9	13,7	-29,2	10,1	-31,2
Fresh water	20	3,5	35,8	15,1	-28,6	6,4	-32,0
Fresh water	20	3,0	35,2	12,4	-28,5	8,1	-30,5
Fresh water	20	3,6	37,0	13,2	-29,2	8,0	-31,5
Saline water	1	3,3	35,3	9,8	-29,2	6,4	-31,4
Saline water	1	3,4	36,3	7,3	-28,7	4,0	-30,1
Saline water	1	2,87	35,29	15,90	-29,06	12,54	-31,76
Saline water	2	3,45	36,71	12,96	-28,57	9,98	-30,16
Saline water	2	3,51	37,49	12,71	-29,41	9,28	-31,35
Saline water	2	3,47	37,23	9,06	-29,57	10,96	-29,95
Saline water	3	3,16	36,64	11,06	-29,35	6,83	-31,50
Saline water	3	3,49	37,71	10,13	-28,92	5,64	-30,92
Saline water	3	3,09	37,26	14,48	-28,53	12,12	-30,01
Saline water	4	3,96	37,63	7,80	-28,89	3,10	-31,42
Saline water	4	3,00	35,04	7,66	-29,55	3,55	-31,68
Saline water	4	3,51	33,48	12,82	-28,60	10,93	-29,55
Saline water	5	2,79	33,19	7,62	-28,93	3,71	-31,53
Saline water	5	2,41	34,58	8,58	-29,60	4,23	-32,21
Saline water	5	2,50	32,76	9,80	-29,59	3,13	-30,11
Saline water	6	3,47	33,61	9,23	-29,28	6,37	-30,30
Saline water	6	3,08	33,67	10,72	-29,63	6,17	-30,86
Saline water	6	2,85	33,16	2,57	-28,85	8,74	-30,81
Saline water	7	3,70	34,57	12,52	-28,20	9,58	-30,46
Saline water	7	3,64	34,48	10,87	-29,05	7,27	-31,45
Saline water	7	3,36	33,94	10,71	-28,81	9,71	-30,03
Saline water	8	2,65	36,51	7,54	-29,82	5,59	-31,30
Saline water	8	3,36	36,92	16,49	-29,94	13,03	-31,26
Saline water	8	2,41	35,83	9,73	-29,39	6,18	-31,91
Saline water	9	2,72	34,23	8,41	-28,81	4,73	-31,26
Saline water	9	3,02	34,22	8,46	-28,42	6,98	-31,96
Saline water	9	3,17	34,37	8,50	-28,82	7,47	-30,93
Saline water	10	-	-	-	-	-	-
Saline water	10	3,98	34,66	14,90	-28,54	13,78	-31,75
Saline water	10	-	-	-	-	-	-
Saline water	11	2,95	35,78	9,14	-29,52	5,64	-31,40
Saline water	11	2,43	26,94	9,00	-29,00	6,19	-31,15
Saline water	11	3,42	35,19	17,62	-27,23	14,03	-28,00
Saline water	12	3,58	34,03	11,20	-28,45	8,40	-30,59
Saline water	12	2,93	34,66	9,61	-29,58	6,96	-31,55
Saline water	12	3,78	34,91	12,44	-28,10	11,06	-30,37
Saline water	13	4,09	35,95	11,97	-28,03	8,59	-29,67
Saline water	13	3,29	37,50	12,20	-29,26	7,85	-30,23
Saline water	13	3,26	35,67	13,97	-28,42	12,03	-31,29
Saline water	14	3,15	35,53	10,65	-28,92	7,05	-31,20
Saline water	14	3,02	32,38	12,89	-28,79	6,65	-30,36
Saline water	14	2,72	36,14	10,47	-28,50	6,48	-30,32
Saline water	15	3,42	33,70	11,82	-29,43	8,60	-31,15
Saline water	15	3,44	35,39	17,25	-29,22	14,27	-30,44
Saline water	15	3,70	35,44	14,75	-29,14	10,71	-30,98
Saline water	16	3,08	32,75	10,56	-29,94	6,68	-33,30
Saline water	16	3,91	35,60	14,30	-28,66	13,21	-30,28
Saline water	16	4,11	35,49	20,41	-28,24	10,79	-30,40

Saline water	17	4,39	36,03	12,02	-28,77	7,79	-29,94
Saline water	17	2,86	33,81	8,54	-28,68	9,34	-31,29
Saline water	17	3,81	37,13	17,49	-28,27	12,70	-29,01
Saline water	18	2,19	28,23	9,19	-26,66	7,53	-29,58
Saline water	18	-	-	-	-	-	-
Saline water	18	-	-	-	-	-	-
Saline water	19	3,35	34,40	9,92	-27,82	6,42	-30,63
Saline water	19	3,20	33,50	9,92	-28,69	8,50	-31,66
Saline water	19	3,03	32,84	11,41	-28,19	11,64	-29,43
Saline water	20	3,10	34,84	8,49	-28,86	4,48	-31,31
Saline water	20	3,64	34,52	12,92	-27,81	8,79	-31,29
Saline water	20	3,19	34,21	10,87	-29,21	10,18	-30,84
